# Prospects of target nanostructuring for laser proton acceleration

**DOI:** 10.1038/srep44030

**Published:** 2017-03-14

**Authors:** Andrea Lübcke, Alexander A. Andreev, Sandra Höhm, Ruediger Grunwald, Lutz Ehrentraut, Matthias Schnürer

**Affiliations:** 1 Max-Born-Institut für Nichtlineare Optik und Kurzzeitspektroskopie, Max-Born-Strasse 2a, 12489 Berlin, Germany

## Abstract

In laser-based proton acceleration, nanostructured targets hold the promise to allow for significantly boosted proton energies due to strong increase of laser absorption. We used laser-induced periodic surface structures generated *in-situ* as a very fast and economic way to produce nanostructured targets capable of high-repetition rate applications. Both in experiment and theory, we investigate the impact of nanostructuring on the proton spectrum for different laser–plasma conditions. Our experimental data show that the nanostructures lead to a significant enhancement of absorption over the entire range of laser plasma conditions investigated. At conditions that do not allow for efficient laser absorption by plane targets, i.e. too steep plasma gradients, nanostructuring is found to significantly enhance the proton cutoff energy and conversion efficiency. In contrast, if the plasma gradient is optimized for laser absorption of the plane target, the nanostructure-induced absorption increase is not reflected in higher cutoff energies. Both, simulation and experiment point towards the energy transfer from the laser to the hot electrons as bottleneck.

Laser driven ion acceleration is an active field of research as it is a promising ion source for basic plasma physics^1^,^2^ and for accelerator technology with special emphasis on biomedical research^3^,^4^. During recent years, different acceleration mechanisms, e.g. target normal sheath acceleration (TNSA), radiation pressure acceleration (RPA) and breakout afterburner (BOA), have been investigated in detail^5^,^6^. In particular for hadron cancer therapy, kinetic energies of several hundred MeV are required and strategies have to be developed on how to possibly reach such energies with femtosecond lasers targeting PW-power (being built at present). Highest proton energies of 93 MeV and above 100 MeV have been reported for RPA and BOA, respectively^7^,^8^. Nevertheless, TNSA from μm thickness targets can be advantageous due to easier implementation of highly repetitive target systems (which will be a necessity when it comes to applications relying on high average flux). Thus, the question is which options for optimizing TNSA are at hand and how they compare with each other. Obviously, the key for optimization is the enhancement of laser absorption and thus the energy transfer from the laser to the ions. One promising idea to reach this goal is the use of nanostructures^9^. Dedicated surface coatings, e.g. micro-particles^10^,^11^, nano-particles^12^,^13^,^14^, bacteria^15^ or foam^14^,^16^,^17^, and surface patterning using heat embossing^18^ or laser structuring^19^ have been applied to study the enhancement of ion energy and flux in laser-ion-acceleration. In those works a beneficial effect of surface structures could be demonstrated: For example, Margarone *et al*. observed an increase of the maximum proton kinetic energy from 25 MeV to 30 MeV and a significant increase of overall proton numbers by use of a nanosphere target^13^, which is still far from the 93 MeV achieved at the very same laser at very similar intensities. In order to enhance the absorption of laser light by nanostructuring, nanostructures of sub-wavelength scale are needed^20^, and it is clear that this approach is applicable to thicker targets, only, i.e. in the frame of TNSA.

Another option for optimizing the TNSA process is enhancing the absorption by a finite plasma gradient. It is known that there is an optimal scale length for laser absorption and proton acceleration if TNSA is active^21^,^22^. Thus, starting from a flat target with step-like density profile, proton acceleration can be optimized by nanostructuring or by a finite plasma scale length. In this work,[Fig f1][Fig f2] we ask the question, under which conditions nanostructuring is beneficial and how nanostructuring compares with the application of an appropriate plasma gradient.

Deriving the plasma scale length from laser parameters is rather complicated. Although usually the contrast between ASE level and main pulse at a given time is provided, it is obvious that it is the full temporal evolution before the peak of the pulse that determines the plasma scale length. For example, the High Field Laser at MBI at which this work has been performed provides a very high contrast even without [Fig f3]a double plasma mirror (DPM) due to its XPW frontend. However, if DPMs are exploited, they will lead to a steepening of the leading edge of the laser. The slower rising edge of the laser pulse without DPM leads to a smaller plasma gradient which improves the coupling of laser energy into the plasma as compared to steeper gradients. This is the reason why for our laser system the DPM is needed for RPA and ultrathin targets but it is not advantageous for the proton acceleration from thicker targets.

In this work, we study the effect of nanostructures on proton acceleration at different laser plasma conditions. Experimentally, we realized this by variation of laser intensity in the range between (5 × 10^17^ … ~10^20^) W/cm^2^. Targets are nanostructured by laser induced periodic surface structures (LIPSS). LIPSS are commonly discussed to be generated by a mechanism based on the interference between the incident laser beam and surface plasmon-polariton waves^23^,^24^ and are intensively studied in respect to a variety of different purposes^24^,^25^,^26^,^27^. Particularly attractive are the typical LIPSS dimensions. In titanium such structures are characterized by a ~600 nm-800 nm periodicity and a height of ~200 nm; the [Fig f4]profile is nearly parabolic (cf. Fig. 5). This is reasonably close to the desirable parameters as determined from theory^19^,^20^. The LIPSS approach allows for very fast and large area surface structuring and yet, it is quite simple and economic. The potential advantage of this technique relies on its *in-situ* implementation. It thus represents a promising nanostructuring technique for highly repetitive target systems in laser ion acceleration.

In this work, we experimentally prove that nanostructuring can significantly enhance laser absorption also at highest intensities and at different plasma scale lengths, i.e. they remain fully functional even at intensities of ~10^20^ W/cm^2^ and without a DPM. Nevertheless, we show that if the laser absorption is optimized by a finite plasma scale length, additional absorption increase due to nanostructuring is not beneficial in terms of maximum ion energy and ion number. However, if the laser plasma conditions are not optimal (i.e. the plasma scale length is too small and/or the intensity is too low), nanostructuring yields strong increase of maximum proton energies and proton numbers.

## Results

Experiments were performed at the Max Born Institute High Field Ti:sapphire Laser. The maximum laser energy on target is ~2 J and the pulse duration is ~35 fs. The laser is focused by a f/2.5 off-axis parabolic mirror to focal spots with FWHM of about 4 μm. The laser contrast is enhanced by a cross polarized wave generation (XPW) front end^28^ and, optionally, a DPM^29^. With DPM, the prepulse-free peak-to-amplified spontaneous emission contrast is better than 10^14^ (ultrahigh contrast), without DPM the contrast is better than 10^10^ (high contrast), which corresponds to ASE intensities of ≤(10^6^ …10^10^) W/cm^2^. Even at the higher ASE intensity without DPM, targets as thin as 30 nm are fully functional and early ablation does not have a significant effect. The reflectivity of the DPM is 80%.

It was our goal to investigate the potential of nanostructures at different laser – target conditions. Experimentally, this was realized by a variation of the focal spot size (by moving the target across the focal region by several hundred μm) at fixed pulse energy and pulse duration as this allows variation of the intensity over a larger parameter range without loosing too much signal. Relevant models of the TNSA mechanism describe the dependence of maximum proton energy as function of laser intensity – no matter whether intensity is changed by varying energy at constant spot size or vice versa^30^,^31^.

To evaluate the intensity, the two-dimensional energy distribution was measured by a microscope at different defocussing conditions. The area at which the laser signal exceeds 1/*e*^2^ of the maximum signal is determined, plotted versus distance from focus and fitted to Gaussian beam propagation model (see Supporting Information). The fit is used to determine the focal area at the defocussing conditions used in the acceleration experiments. Depending on the particular focus conditions, typically between 30% and 50% of the total laser energy is confined within the focal area, where this ratio tends to become larger as the beam is stronger defocused. We calculated the intensity assuming 50% of the energy being confined in the focal area. The pulse energy was measured for every shot by a calibrated leakage through a mirror. Targets are irradiated at normal incidence with intensities up to 8 × 10^19^ W/cm^2^. The correspondent peak intensity is ~1 × 10^20^ W/cm^2^.

**Figure 1 f1:**
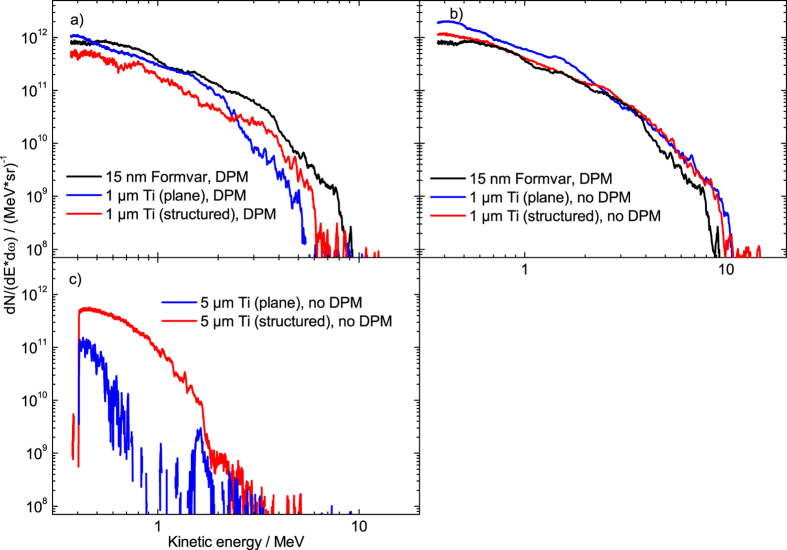
(**a**) Proton spectra from different targets obtained at a laser intensity of 5 · 10^19^ W/cm^2^ with DPM; (**b**) same as (**a**) but without DPM and slightly higher intensity of 8 · 10^19^ W/cm^2^ for titanium foils (**c**) Same as (**b**) but for 5 μm thick targets and laser intensity of 8 · 10^17^ W/cm^2^. The background has been subtracted.

Highest proton energies were so far obtained with ultrathin films and ultrahigh laser contrast. Since the laser plasma parameters are subject to (small) variations on a daily base, all data which we directly compare are collected at the very same day. One example of a proton spectrum from an ultrathin formvar film is shown in Fig. 1a. In this specific case, the maximum energy obtained with 15 nm Formvar is (8.6 ± 0.3) MeV. In the same graph we show the proton spectra obtained from 1 µm thick, plane and nanostructured titanium foils. In agreement with others^10^,^11^,^12^,^13^,^14^,^15^,^16^,^17^,^18^ we observe a positive effect of the nanostructures on the maximum proton energies, which increase from 5.3 MeV to 6.1 MeV, i.e. by 15%. Since for these thicker targets the use of a DPM is no longer advantageous and reduces the available energy on target by 20%, in Fig. 1b we also compare the RPA spectrum with spectra obtained from plane and nanostructured Ti foils without DPM. In this case all three spectra are very similar and we do not observe any beneficial effect of the nanostructures. Apparently, the XPW front-end realizes sufficiently large plasma gradients to efficiently accelerate protons from 1 μm thick titanium foils and leads to as high cutoff energies as those obtained from RPA of a 15 nm thin plastic foil.

From the comparison to RPA measurements and dedicated scans^32^ we conclude that we have performed our experiments at nearly optimal laser parameters. The situation becomes very different if we deviate from the optimum laser-target parameters, i.e. reduce the laser intensity. In Fig. 1b we show the example of plane and nanostructured 5 µm thick titanium foil irradiated at reduced intensity (8 · 10^17^ W/cm^2^). Under these conditions, we observe a huge effect of nanostructuring on the maximum proton energies. These are 0.7 MeV in the plane target case, but 1.8 MeV in the nanostructured case, which corresponds to an increase by a factor of 2.6. The impact of nanostructuring on the efficiency of energy transfer from laser energy into proton kinetic energies (>0.4 MeV) is even more pronounced: here we observe a 17-fold increase.

**Figure 2 f2:**
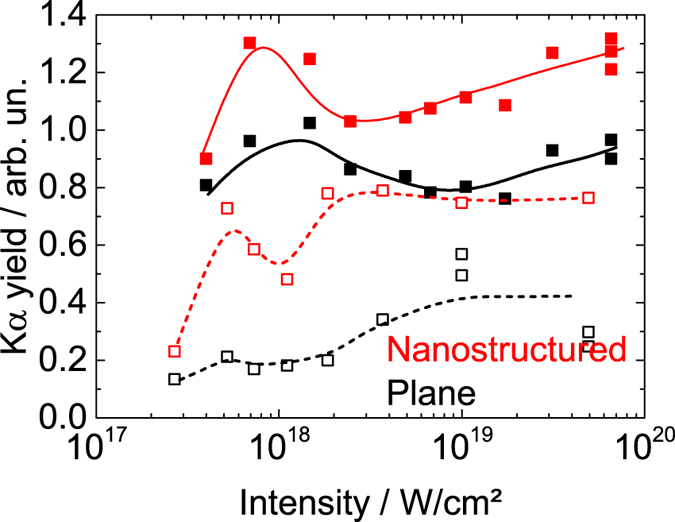
K*α* yield as function of intensity for structured (red) and plane (black) target with (open symbols, broken line) and without (filled symbols, solid line) DPM. Angle of laser incidence is about 30°. Symbols represent experimental data, lines serve as guides to the eye.

In order to exclude a destruction of the nanostructures at highest intensities by a prepulse or the ASE level and to probe the effect of nanostructures on the laser absorption we investigated the K*α* yield. K*α* emission is strongly dependent on the electron numbers and their energy distribution function and is thus a sensitive measure of laser absorption^33^. In Fig. 2 we demonstrate enhanced emission from nanostructured samples both, with and without DPM, and for a wide intensity range. For both plasma scale lengths, we observe a significant increase of K*α* due to the surface structuring. Regarding the contrast conditions, significantly larger K*α* yields are observed if no DPM is used. These results clearly show that the nanostructures are functional in both contrast conditions, i.e. laser absorption is significantly enhanced in the presence of nanostructures – even at highest intensities. We note that this enhanced absorption may not necessarily originate from the original structure but possibly from a dynamically generated replica of the structure which is formed as a result of plasma collisions^19^.

Nevertheless, the more efficient laser–target coupling is not reflected in the proton spectrum (Fig. 1b) at highest intensities. This indicates that an increase of absorption does not immediately lead to higher proton energies, an issue that will be discussed in more detail in the discussion section.

**Figure 3 f3:**
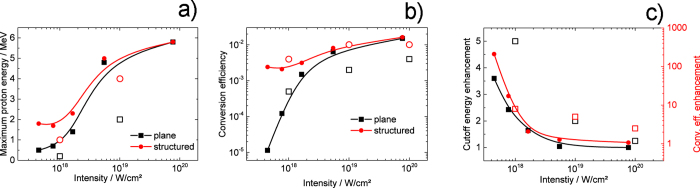
Intensity-dependent maximum proton energies (**a**) and efficiency of energy conversion from laser energy into proton kinetic energies >0.4 MeV (**b**) for plane and nanostructured target from experiment (filled symbols) and simulation (open symbols). The relative enhancement of energy conversion efficiency (red) and maximum proton energy (black) due to nanostructuring from experiment (filled symbols) and simulation (open symbols) is shown in (**c**). Lines serve as guide to the eye. Please note, the simulation was carried out for a 1 m thick Ti foil and an ultrahigh contrast, while a 5 μm foil at high contrast conditions was used in experiment.

The results of a more systematic study are shown in Fig. 3a–c. As expected, we observe a strong increase of cutoff energy with intensity (Fig. 3a) for both the plane and the nanostructured target. For the nanostructured target we observe higher cutoff energies than for the plane target, but this positive effect of the nanostructure is a function of intensity. While at ~4 × 10^17^ W/cm^2^ LIPSS increases the cutoff energy by a factor of 3.6, the effect becomes negligible above ~5 × 10^18^ W/cm^2^ for our target (Fig. 3c). The energy conversion efficiency *η* (Fig. 3b,c) presents a similar picture. Here, we define it as 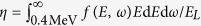, where the lower integration limit is determined by the low-energy cutoff of the spectrometer, *f(E, ω*) is the spectral distribution at the solid angle *ω* and *E*_*L*_ is the laser energy incident on the target. An effective emission cone of *ω* = 0.2 sr is assumed^34^. The conversion efficiency increases strongly with intensity for the plane target case reaching a value of 1.5% at 8 × 10^19^ W/cm^2^ which is in agreement with the highest reported conversion efficiencies obtained with a single laser pulse so far^32^,^35^,^36^,^37^. For nanostructured targets the conversion efficiency is always larger, but its intensity dependence is significantly weaker than for the plane target. At highest intensities both target systems yield very similar conversion efficiencies. From these results we can immediately draw two important conclusions: First, at intensities where the rising pulse front can not establish a density gradient at the surface enabling efficient light absorption (in our case lower intensities), nanostructuring leads to a huge enhancement of conversion efficiency by up to a factor of 200 and an enhancement of cut-off energy by up to a factor 4. Second, at intensities at which the rising pulse generates a significant change of the plasma density gradient (in our case highest intensities), nanostructuring does not provide any further positive effect on the conversion efficiency. We stress that our data on K*α* emission clearly show that even if a finite density gradient is generated by the leading edge of the laser pulse the nanostructures remain functional.

## Theory

The results are compared to particle-in-cell (PIC) simulations at different intensities (open symbols in Fig. 3), for which the two-dimensional modified PSC code^38^ was used. The simulation box (15000 × 5000 cells) is 30 × 25 µm^2^ large with step sizes of 2 nm in longitudinal and 5 nm in transversal direction. The time step is 2 nm/*c*, and 30 particles per cell were considered. The simulation is collisionless and used periodic boundary conditions. A p-polarized ultrahigh contrast laser pulse (35 fs, 4 μm spot size, super-Gaussian/Gaussian in space/time, no pre-pulses) interacting with a Ti target of density 6 × 10^22^ cm^−3^ is considered. This corresponds to an ultrahigh contrast case. An average ionization state <*Z*> = 10 at I_*L*_ = 10^19^ W/cm^2^ is evaluated from the Ammossov-Delone-Krainov (ADK) model^39^,^40^. The target thickness is limited to 1 μm for numerical reasons, which is still a sufficiently good approximation of the experimental case. The total simulation time is 300 fs. The nanostructured target is simulated by a parabolic relief of 200 nm height and 600 nm period. The contamination layer at the target rear side serving as particle reservoir for the acceleration process was modelled by a 40 nm thick layer of C^5+^H^+^ at solid density. The acceleration of light ions in the field of heavy ions depends on the ion density ratio. Best agreement with experimental data is found for 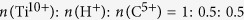. With these input parameters the simulation can qualitatively explain the general trend of the intensity dependence of conversion efficiency and enhancement of cutoff energy (Fig. 3b,c). We generally observe a larger effect of the nanostructures on the conversion efficiency in the simulation than in the experiment, which is not surprising: Our targets are commercially available Ti foils which are not optically flat but possess a microstructured surface (fine grooves due to manufactering) which increases the energy coupling from the laser to the target. A larger discrepancy between experiment and simulation is observed for the cutoff energy. At highest intensities, the simulations yield a factor ~2 higher cutoff energies than the experiments. There are several possible reasons for this: Firstly, it is well known that 2D simulation overestimate maximum proton energies by a factor 2–3^41^,^42^. Secondly, the target thickness in the experiment was 5 μm, while in the simulation it was only 1 µm. At lower intensities the maximum proton energies obtained from simulation are in much better agreement with experiment. This is because we experimentally vary the intensity by changing the focal size which makes the experimental geometry at lower intensities, i.e. larger focal spot sizes, closer to two-dimensional.

## Discussion

At low intensities, surface modifications change the absorbance by different mechanisms^43^. Surface roughness can enhance the absorption of light by multiple reflections in microcavities and by variation of the incidence angle. In nanostructures the absorbance is further altered since the optical properties are different from bulk material. Furthermore, LIPSS can enhance the laser absorption by generation of surface electromagnetic waves. Vorobyev has shown that LIPSS are composed of a multidude of differently sized structures ranging from ~10 nm to μm scale^43^ wich is also reflected in our TEM images (not shown here). Stephens and Cody have developed a theory to describe the effect of deep-subwavelength surface structures on the reflectivity^44^. The main effect is a gradual change of dielectric function from vacuum to bulk value, which leads to significantly reduced reflection of light of wavelength several times the characteristic scale length. For example, in titanium with a linear mass grade of 100 nm they calculated a decrease of reflectivity at 800 nm from about 60% for the plane surface to 40% for the textured surface, which corresponds to an increase of absorption by 50%. This anti-reflection mechanism is intriguingly similar to the function of a plasma gradient for laser absorption at ultra-high intensities. One can therefore see the role of deep-subwavelength nanostructures in laser plasma interaction as mimicking a finite plasma gradient. In our case, the density scale lengths imposed by the nanostructures (~ structure heigth, i.e. ~200 nm) and plasma gradient at highest intensities without DPM (a scale length of 400 nm is obtained from hydrodynamic calculations using our experimental third order autocorrelation trace) are quite similar.

In the experiments, two different plasma gradients were realized by using or omitting the DPM. We observe a significant K*α* yield increase due to nanostructuring for both plasma gradients. The K*α* yield from the structured and plane target at lower plasma gradient (no DPM) is almost a factor 2 higher than for the respective target at large gradient. On the one hand, this shows clearly that the structures are functional also in case of the lower plasma gradient (larger scale length). On the other hand it brings up the question *why* both beneficial effects can be combined. We suggest that this is due to the significantly larger surface area in the structured case than in the plane case. Thus, the plasma gradient increases the absorption, the nanostructures, additionally, increase the surface, i.e. more electrons can be accelerated and generate characteristic X-radiation. The reader may remark that at highest intensities the ASE level or the leading edge of the pulse is sufficiently intense to generate a plasma that fills the structure gaps and increases the laser absorption as well as the X-ray yield. However, we exclude such a surface levelling because the plasma streams off the surface perpendicular which will lead to plasma collisions and stagnation, i.e. the formation of stable secondary structures^19^,^45^. Those structures will survive for several ns.

Regarding proton acceleration, our results show that nanostructured surfaces can have a positive effect on the energy transfer from laser to protons and on the maximum kinetic energies in TNSA. However, this is only true for conditions that are far from optimum, i.e. where the laser absorption is not optimized by an appropriate density gradient. This is the case for ultrahigh contrast laser pulses or too low laser intensities. In case of nearly optimal conditions, neither the energy transfer nor the maximum kinetic energy of the protons can be further increased within experimental uncertainty. Margarone *et al*. and Prencipe *et al*. have observed a beneficial effect of the structured target even at higher intensities than used in our experiments^13^,^17^. However, for the plane target Margarone *et al*. report energy conversion efficiencies of 0.3% for protons with energies above 5 MeV and a cutoff energy of 24 MeV. In particular, the relatively low conversion efficiency indicates that their laser – target system is not optimal. Indeed, the laser contrast used in their experiments (3 × 10^11^) and, in particular, the steep leading edge of the laser pulse provided by the DPM, are far from being optimal for TNSA. For RPA, on the other hand, their target thicknesses is too large. Consequently, they have observed a strong enhancement effect. Plane targets of Prencipe *et al*. had very similar thicknesses as Margarone’s and their experiments were performed at the very same laser system, i.e. also in that work the plane target was not optimal. This becomes especially evident if their results are compared to the maximum proton energies reached at that laser system (93 MeV), which were obtained from 15 nm thin foils^7^. In fact, with the use of a DPM, we could qualitatively reproduce these results: In agreement with Margarone *et al*., LIPSS can increase the cutoff energy by 15% for ultrahigh contrast. But the resulting cutoff energies of 6.1 MeV (for the structured target) is still significantly lower than the cutoff energies obtained at optimal laser target conditions, i.e. with ultrathin films or with thicker targets at high contrast.

### How can these seemingly contradictory results on Kα yield and proton acceleration be understood?

Our K*α* yield measurements clearly show that nanostructuring increases the energy transfer from the laser to electrons. Nevertheless, this additional energy is not reflected in higher proton energies if the laser plasma coupling is optimized by a plasma scale length. Increased absorption will be reflected in the electron energy distribution function by an increased number of electrons and/or by an increased average energy of the electrons. As we have discussed already above, we expect that the increased absorption mainly leads to an increased number of electrons. Proton acceleration in the TNSA mechanism is mainly due to electrons with energies >0.1 MeV. Our K*α* measurements do not allow to draw conclusions about possible change of electron energy distribution function, i.e. it is conceivable that the hot electron number and, in particular, temperature does not change, significantly. The proton cutoff energy is mainly given by the hot electron temperature and does only weakly depend on their number. Although the laser absorption is increased by the nanostructures even in case of appropriate plasma gradient, this does not lead to a significant increase of cutoff energy, if the hot electron temperature is not increased. Thus, our data indicate a limit in the energy transfer from the laser to the hot electrons.

**Figure 4 f4:**
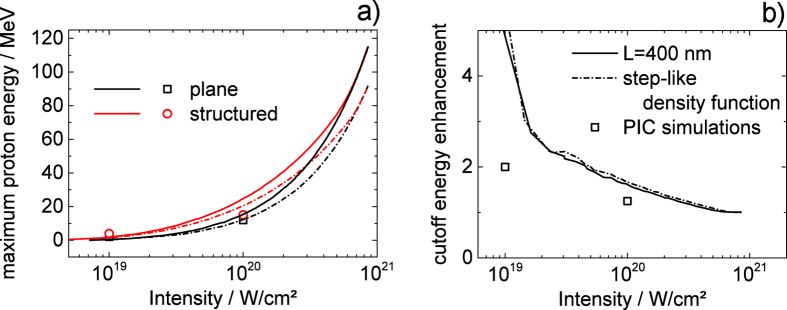
(**a**) Maximum proton energies as function of laser intensity for plane (black) and structured (red) target with step-like density profile (dashed lines) or at plasma scale length *L* = 400 nm (solid lines) obtained from combined PIC simulation (step-like density profile only) and analytical model (cf. Supp. Information) (**b**) Proton energy enhancement due to nanostructuring as function of intensity for the two different laser plasma conditions. The two curves in (**b**) show the ratio of the red and the black curves in (**a**). Symbols are results from PIC simulation as shown in Fig. 3.

We have also performed calculations to investigate the use of nanostructuring at even higher intensities. Andreev *et al*. have shown that electron acceleration is enhanced and the hot electron energy is increased for a structured target^9^. The basic idea behind their model is that electrons are ripped out of structure’s side walls and experience additional “vacuum heating”. It is obvious that for a given inter-wall spacing there is a limit of electron kinetic energy - at a certain laser intensity the excursion length of the electron in the laser field will exceed the inter-wall spacing and electrons will enter the opposite wall and thereby leaving the laser field. Of course, the inter-wall distance may be increased but even in this case the maximum possible electron energy will rapidly converge because the share of the side wall surface in total target surface will become small. Model calculations were performed for two different targets (plane target and structured target) at two different laser contrast conditions (step-like density profile and finite scale length *L* = 400 nm) to compare the resulting maximum proton energies (for details see the Supp. Information). The results are shown in Fig. 4. From these calculations we derive a couple of important conclusions: (1) At high intensities (here: ~6 × 10^20^ W/cm^2^) the beneficial effect of the structure in terms of maximum proton energy vanishes for both contrast conditions. (2) The maximum proton energy obtained with a given target at a finite scale length is always larger than for the step-like density profile. (3) There is a certain intensity, above which the plane target at a certain plasma scale length even outperforms the structured target with a step-like density profile. Of course, we should stress the point that these conclusions only hold if the target thickness is sufficiently large to grant the functionality of the target even for the finite plasma scale length. As evident from a comparison of Fig. 4b) and 3c), theory and experimental results are in qualitative agreement.

In conclusion, we have shown that LIPSS nanostructures significantly enhance laser absorption also at finite plasma gradients and intensities up to 10^20^ W/cm^2^. The nanostructures remain functional even at our highest intensities and the given plasma gradient. In case the laser absorption was already optimized for proton acceleration by an appropriate plasma gradient, additional surface nanostructuring did not further increase the maximum proton energies. Since it is the hot electron temperature that mainly determines the proton cutoff energy, this observation indicates a fundamental limitation in energy transfer processes which inhibits a significant increase of hot electron average kinetic energy beyond that obtainable with optimized laser–plane target systems. Nevertheless, we have clearly shown that for non-optimized laser–target systems, nanostructuring can significantly improve proton acceleration in terms of maximum energy and proton numbers. For example, nanostructuring will be very helpful if the given laser intensity and contrast conditions result in low absorption due to a step-like density gradient. Furthermore, LIPPS – a laser based surface structuring method – provide close to optimum structural parameters for enhanced optical laser light absorption. This structuring method is easy to implement for any laser driven secondary source, works robust, *in-situ* and can be applied on a millisecond basis in between successive high power laser shots.

## Methods

**Figure 5:**
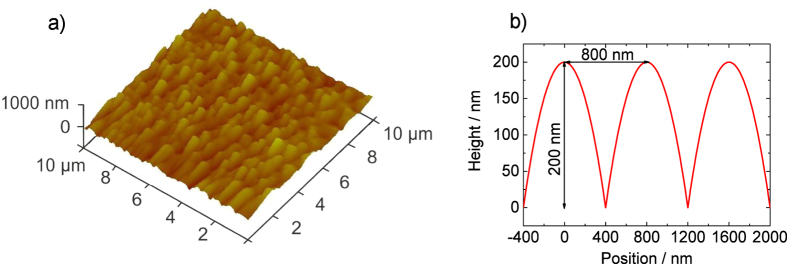
(**a**)AFM image of a laser structured 5 μm thick titanium foil. (**b**) Approximated parabolic profile of the structure.

To nanostructure our targets we used an *in-situ* method to generate laser induced periodic surface structures (LIPSS). By *in-situ* we mean that the sample is nanostructured in the very same setup which is used for the high-intensity shots, with the same, but strongly attenuated laser. In practice, the target is carefully aligned with respect to focal conditions, then the laser beam diameter is reduced from ~60 mm to ~6 mm by an aperture. The laser energy on target is further reduced to 10–30 μJ and typically ~20 pulses are applied to the target surface for LIPSS generation. The applied fluence is 2–6 J/cm^2^. Immediately after structuring and without change in the alignment, the aperture is opened again and a full power laser pulse is applied. The laser illumination parameters for LIPSS generation were carefully investigated in a separate study and resulting nanostructures were characterized by scanning electron microscopy (SEM) and atomic force microscopy (AFM). A typical AFM image of LIPSS structure obtained with the same illumination conditions as used for the proton acceleration experiments is shown in Fig. 5. For titanium foils we typically obtain a ripple structure with ~600 nm–800 nm periodicity and ~200 nm height in agreement with others groups^46^,^47^.

Accelerated particles are detected in single-shot experiments by a Thomson spectrometer coupled to a MCP in laser propagation direction. The spectrometer entrance orifice has a diameter of 200 μm and it is placed at 70 cm distance from the target.

For studying the effect of LIPSS on the K*α* emission, we have introduced a plane silicon (111) crystal into the setup as a simple x-ray spectrometer. For these studies, the angle of incidence is not any longer 0° but the target was rotated by about 30° to enhance the K*α* yield at lower intensity by bringing Brunel heating into action. Furthermore, the target thickness is increased to 5 μm. This is still significantly smaller than the Ti K*α* attenuation length in titanium (~20 μm). X-rays emitted from the target front side were detected. The diffracted photons are recorded by a CCD camera and measured as function of intensity for plane and nanostructured targets at high and ultra-high contrast. The intensity was varied by varying the laser spot size, keeping laser pulse energy and pulse duration constant.

## Additional Information

**How to cite this article:** Lübcke, A. *et al*. Prospects of target nanostructuring for laser proton acceleration. *Sci. Rep.* **7**, 44030; doi: 10.1038/srep44030 (2017).

**Publisher's note:** Springer Nature remains neutral with regard to jurisdictional claims in published maps and institutional affiliations.

## Supplementary Material

Supplementary Informatison
