# Supplementary material for: Prospects of target nanostructuring for laser proton acceleration
Source: Sci Rep. 2017 Mar 14;7:44030. doi: 10.1038/srep44030 (PMC5349587; doi:10.1038/srep44030)
Supplement: Supplementary Informatison [file srep44030-s1.pdf]

# Prospects of target nanostructuring for laser proton acceleration

Andrea Lübcke, Alexander A. Andreev, Sandra Höhm, Rüdiger Grunwald, Lutz Ehrentraut, M. Schnürer

Max-Born-Institute for Nonlinear Optics and Short Pulse Spectroscopy,
Max-Born-Strasse 2A, 12489 Berlin, Germany
luebcke@mbi-berlin.de

# 1 Analytical model of maximum proton energies

Since PIC simulations are time-consuming and costly, they are restricted to certain target–laser situations. Target–laser interactions that would require substantially more extensive simulation time, e. g. to account for the effect of a finite plasma scale length or the effect of a surface structure are examined in the frame of an analytical model which is calibrated with a simulation. The maximum proton energy obtained in TNSA is given by the average hot electron temperature and the electron density [1]:

$$E_{\max} \simeq 2E_0 \left[\ln(2\tau)\right]^2 , \quad (1)$$

with

$$E_0 = Z k_B T_h \quad (2)$$

$$\tau \simeq \frac{\omega_{pi} t_L}{\sqrt{2e}} \quad (3)$$

$$\omega_{pi} = \sqrt{\frac{n_{e0} Z e_0^2}{m_i \epsilon_0}}, \quad (4)$$

where $Z$ is the ion charge, $k_B$ is Boltzmann's constant, $T_h$ is the hot electron temperature, $t_L$ is the laser pulse duration [2], $e_0$ is elementary charge, $e$ is Euler's number, $n_{e0}$ is the electron density in the unperturbed plasma, $m_i$ is the ion mass and $\epsilon_0$ is the vacuum permittivity. Thus, the key to the maximum proton energy is the electron energy distribution function (EDF) that provides both, the hot electron temperature and the electron density. For relativistic laser intensities, the EDF can me modelled as sum of non-relativistic ($T_c \ll m_e c^2$) and ultra-relativistic ($T_h \gg m_e c^2$) Maxwellian energy distribution functions with temperatures $T_{c,h}$ and electron numbers $N_{c,h}$, where the indices refer to *cold* and *hot* [3]:

$$f_e(E) = 2N_c \sqrt{\frac{E}{\pi T_c^3}} \exp\left(-\frac{E}{T_c}\right) + N_h \frac{E^2}{2T_h^3} \exp\left(-\frac{E}{T_h}\right) . \quad (5)$$

Electron numbers and temperatures depend on laser and target conditions and were obtained from conservation laws:

$$T_h \approx T_{0h} \left(\sqrt{1+\eta I_{18}} - 1\right) \quad (6)$$

$$T_c \approx \frac{m_e c^2 \eta^2 \epsilon_L^2}{2N^2 T_h^2} \quad (7)$$

$$I_{18} = \frac{I_L \lambda_L^2}{1.37 \cdot 10^{18}} (\mathrm{W \mu m^2/cm^2}) \quad (8)$$

$$N_h = N \frac{\sqrt{T_c/m_e c^2}}{1+\sqrt{T_c/m_e c^2}} \quad (9)$$

$$N_c = \frac{N}{1+\sqrt{T_c/m_e c^2}} \quad (10)$$

$$N = \frac{\eta \epsilon_L \left(1+\sqrt{T_c/m_e c^2}\right)}{T_c + T_h \sqrt{T_c/m_e c^2}}, \quad (11)$$

where $\lambda_L$ is the laser wavelength, $m_e$ is the electron mass, $I_L$ and $\epsilon_L$ are laser pulse intensity and energy, respectively and $c$ is the speed of light. The share of absorbed energy in incident laser energy is

$$\eta \approx \chi_{\mathrm{struc}}(I) \eta_p(I), \quad (12)$$

where $\chi_p(I)$ is the absorption coefficient for a plane target with step-like density profile [4]. The contribution from the structure is described by

$$\chi_{\text{struc}} = \begin{cases} 1 & \text{plane target} \\ 1 + \frac{d_1/2l_{\text{extr}}}{\sqrt{1+(d_1/2l_{\text{extr}})^2}} \cdot \frac{d_2/2r_{eh}}{\sqrt{1+(d_2/2r_{eh})^2}} \cdot \frac{2h}{(d_1+d_2)} \cdot \frac{4\pi r_{eh}}{\sqrt{2}\eta c t_L} & \text{struc. target} \end{cases},$$

where in this particular case a rectangular profile was assumed with $d_1$ being the side wall width, $d_2$ being the inter-wall distance and $h$ being the height of the relief. $t_L$ is the laser pulse duration, $r_{eh} \approx eE_L/m_e\omega_L^2$ is the characteristic size of an electron orbit in vacuum due to its motion in the laser field $E_L$, $\omega_L$ is the laser frequency, $l_{\text{extr}} = E_L/en_e$ is the extraction depth, i. e. the depth from which the laser field can pull electrons into vacuum, and $n_e$ is the initial electron density. For finite plasma scale lengths $L$, the absorption is further enhanced by $\chi_L \approx 1 + 0.6L/\lambda$ [3, 5]. This analytical model is calibrated with a PIC simulation for a step-like density profile, i. e. the calibration temperature $T_{0h}$ is taken from the simulation. For the calculations shown in Fig. 4 of the manuscript, PIC simulations were carried out for a 1 μm thin solid-density $Si^{14+}$ target and a 33 fs long laser pulse. In the simulation a parabolic profile (period: 600 nm, height: 200 nm) was used, but in the analytic description, this parabolic profile was approximated by a rectangular profile with $d_1 = d_2 = 300$ nm and $h = 150$ nm.

# 2 Evolution of focal spot size with defocusing

Our laser beam has a non-ideal pulse shape and the intensity distribution at a given distance from the focus is not Gaussian. As already briefly explained in the manuscript, we have measured the two-dimensional intensity distributions at different distances from the focus with a CCD camera. The pixel with highest intensity was identified, and all pixels with intensity increasing $1/e^2$ of the maximum intensity were counted, yielding the $1/e^2$ focal area. An average focal size as given by the square root of the focal area is plotted in Fig. 1 and can nicely be fitted to the Gaussian beam propagation model. If there were significant hot spots in the profile, the measured beam sizes would significantly deviate from the model. This is not the case.

# References


[1] P. Mora. Plasma expansion into a vacuum. *Phys. Rev. Lett.*, 90:185002, 2003.

[2] J. Fuchs, P. Antici, E. D'Hummiéres, E. Lefebvre, M. Borghesi, E. Brambrink, C. A. Cecchetti, M. Kaluza, V. Malka, M. Manclossi, S. Meyroneinc, P. Mora, J. Schreiber, T. Toncian, H. Pépin, and P. Audebert. Laser-driven proton scaling laws and new paths towards energy increase. *Nature Phys.*, 2:48, 2006.

[3] Alexander Andreev. *Theory of Laser-Overdense Plasma Interactions*, pages 19–45. Springer International Publishing, Heidelberg, 2013.

[4] P. Gibbon, A. A. Andreev, and K. Y. Platonov. A kinematic model of relativistic laser absorption in an overdense plasma. *Plasma Phys. Control. Fusion*, 54:045001, 2012.

[5] A. A. Andreev and K. Y. Platonov. Hard x-ray generation and particle production via the relativistic-intensity laser pulse interaction with a solid target. *Laser Part. Beams*, 18:81, 2000.

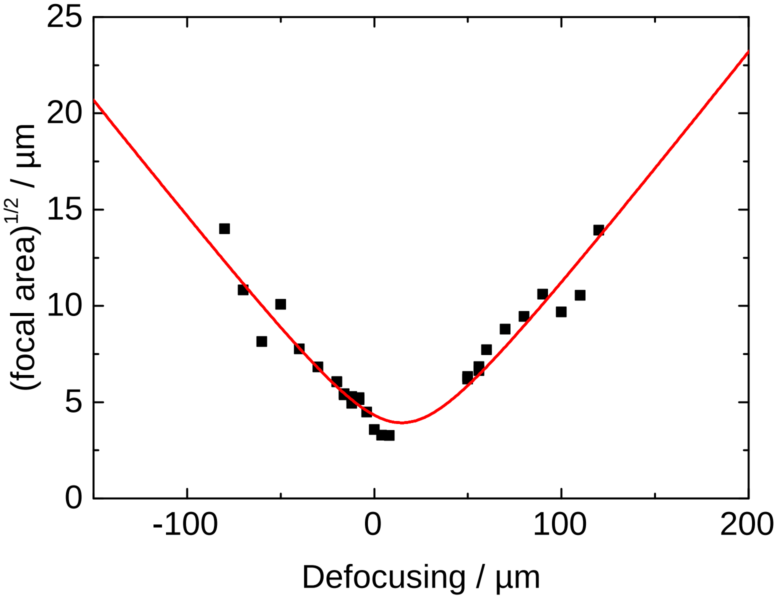


Figure 1: Experimental spot size evolution (data points) and fit to Gaussian beam propagation model (line).
